# High-grade glioma with pleomorphic and pseudopapillary features: a single-institution series of three cases

**DOI:** 10.1186/s40478-025-02097-7

**Published:** 2025-08-18

**Authors:** Eric A. Goethe, Rasha Alfattal, Subhiksha Srinivasan, Pushan Dasgupta, Vinay Puduvalli, Shiao-Pei Weathers, Leomar Y. Ballester, Jeffrey S. Weinberg, Sujit Prabhu, Sherise D. Ferguson, Maria A. Gubbiotti

**Affiliations:** 1 Department of Neurosurgery, The University of Texas MD Anderson Cancer Center, Houston, TX 77030 USA; 2 Department of Neurosurgery, Baylor College of Medicine, Houston, TX 77030 USA; 3 Division of Pathology and Laboratory Medicine, University of Texas MD Anderson Cancer Center, Houston, TX 77030 USA; 4 Department of Neurosurgery, McGovern Medical School, Houston, TX 77030 USA; 5 Department of Neuro-oncology, University of Texas MD Anderson Cancer Center, Houston, TX 77030 USA

## Abstract

**Introduction:**

Modern molecular diagnostic techniques such as DNA methylation profiling are leading to the reclassification of several central nervous system malignancies and discovery of novel diagnostic entities, such as high-grade glioma with pleomorphic and pseudopapillary features (HPAP).

**Methods:**

We performed a retrospective chart review of all patients with HPAP confirmed with methylation profiling at a single institution between 2023 and 2025. Demographic, radiographic, surgical, and outcome data were collected.

**Results:**

Three patients were identified: two females and one male with a mean age of 49.7 years (range 25–62). No patients had a prior cancer history. One patient had an incidentally discovered tumor, while the other two patients underwent imaging for symptoms of headache, vision changes and extremity weakness. Mean tumor size was 4.0 cm (range 2.8-6) with a wide variation in imaging characteristics. All patients underwent surgical resection and radiographic gross total resection was achieved in all cases. All patients underwent radiation therapy without concurrent chemotherapy. After a median 20 months follow up (range 4.5 to 108.9), two patients experienced tumor progression at 2.7 months and 86.5 months respectively. All patients were alive at last follow up.

**Conclusion:**

HPAP is a novel clinical entity demonstrating variable molecular signatures sharing a common DNA methylation profile which demonstrates a relatively favorable clinical course when compared with other high grade gliomas. Further study is needed to determine the optimal treatment and factors that influence survival.

## Introduction

While the classification of central nervous system (CNS) tumors has been historically based on histological features and certain widely known molecular findings, modern diagnostic techniques, including more sophisticated next generation sequencing (NGS) and DNA methylation profiling, are leading to the reclassification of many tumors. For example, high grade astrocytoma with piloid features (HGAP) is one such entity, first described in 2018 and the first CNS tumor to require methylation profiling for diagnosis [1]. Another example is the embryonal tumor with multilayered rosettes (ETMR), an amalgamation of embryonal tumor with abundant neuropil and true rosettes (ETANTR), ependymoblastoma, and medulloepithelioma [2]. 

Of clinical interest, there are reports of patients with glioblastoma, IDH-wildtype (GBM) with long survival times [3]. In 2023, Pratt et al. [4] published the first report about a class of circumscribed gliomas, high grade glioma with pleomorphic and pseudopapillary features (HPAP), that demonstrate frequent chromosome 13 loss with a unique characteristic methylation profile. Importantly, despite sharing some features with GBM, these neoplasms show better clinical outcomes and may represent a fraction of GBM with long-term survival. Given the more optimistic prognosis in this tumor type and the sparse available literature regarding HPAP, we performed our own institutional investigation in order to better characterize the radiologic, histomorphologic, and clinical course of this novel tumor type.

## Methods

We searched the electronic medical records of all patients with confirmed HPAP treated at our institution between 2023 and 2025 for demographic, clinical, radiographic, surgical, pathological, and outcome data. Demographic data included age at time of surgery, sex, and race. Clinical data included personal cancer history, presenting symptoms, functional status as defined by Karnofsky performance score (KPS), and postoperative radiation and systemic treatment data. Radiographic data was based on preoperative magnetic resonance imaging (MRI) and included tumor size, enhancement pattern, and presence of leptomeningeal dissemination. Surgical data included extent of resection. Pathological data included histologic diagnosis, mutational data, and results of molecular and epigenetic testing.

Next generation sequencing was performed at three separate institutions. Case 1 underwent testing using the Mutation Analysis Precision Panel (MAPP) test at MD Anderson Cancer Center. This panel is a custom high-throughput next generation sequencing-based CLIA assay that uses targeted hybridization-based capture technology for detection of sequence variants/mutations in 610 genes (single nucleotide variants [SNVs] and insertion/deletion alterations [indels]), copy number variants (CNVs) in 583 genes, select gene rearrangements (Fusions) in 34 genes, and selected genomic immuno-oncology signatures including microsatellite instability (MSI) and tumor mutational burden (TMB) in DNA isolated from formalin-fixed paraffin embedded (FFPE) tumor tissue and cytology specimens. The assay employs DNA extracted from both tumor tissue and paired normal (blood or tissue) specimens in a CLIA-certified molecular diagnostic laboratory. A minimum of 50 ng of genomic DNA undergoes whole-genome library construction with adapters carrying Unique Molecular Indices (UMI) allowing tagging of original double-stranded DNA that facilitates statistical reconstruction of reads sequenced as duplicates from a single-amplified genome. A target area of 2.1 megabases (hg19 genome) is enriched with custom hybrid-capture, 120nt dsDNA probes. The assay uses the NovaSeq 6000 next generation sequencing platform and bidirectional paired-end sequencing to identify nucleic acid variants for all coding regions from most genes in the panel, the TERT promoter, 1 non-coding RNA gene, and clinically relevant rearrangements. Reported somatic mutations are identified by comparison to the human genome reference sequence GRCh37/hg19 and reviewed in OncoSeek against a process-matched normal control. Data analysis is performed in house by a bioinformatics pipeline which relies on the dual-duplex molecular barcoding for consensus analysis to reduce sequencing artifacts and achieve greater sensitivity and positive predictive value.

### Case 2

underwent analysis using the University of Pittsburgh Medical Center’s GlioSeq, an amplification based targeted NGS of DNA and mRNA which can detect SNVs, small insertions/deletions in targeted regions of 47 genes, copy number changes in 43 genes, and structural alterations involving 103 genes performed on FFPE specimens.

### Case 3

was analyzed via Neogenomics Neotype DNA and RNA assay which covers 50 genes to include SNVs/InDels for 29, CNV for 3, and fusion testing for 34 genes. FISH (also performed at Neogenomics) was used to assess changes in chromosomes 7 and 10. All cases underwent DNA methylation profiling at the NIH using versions 11b6 and 12b6 of the Heidelberg classifier as well as versions 2.0 or 3.0 of the NCI/Bethesda classifier [4].

Outcome data included progression-free and overall survival from time of surgery.

### Case narratives

#### Case 1

**Fig. 1:**
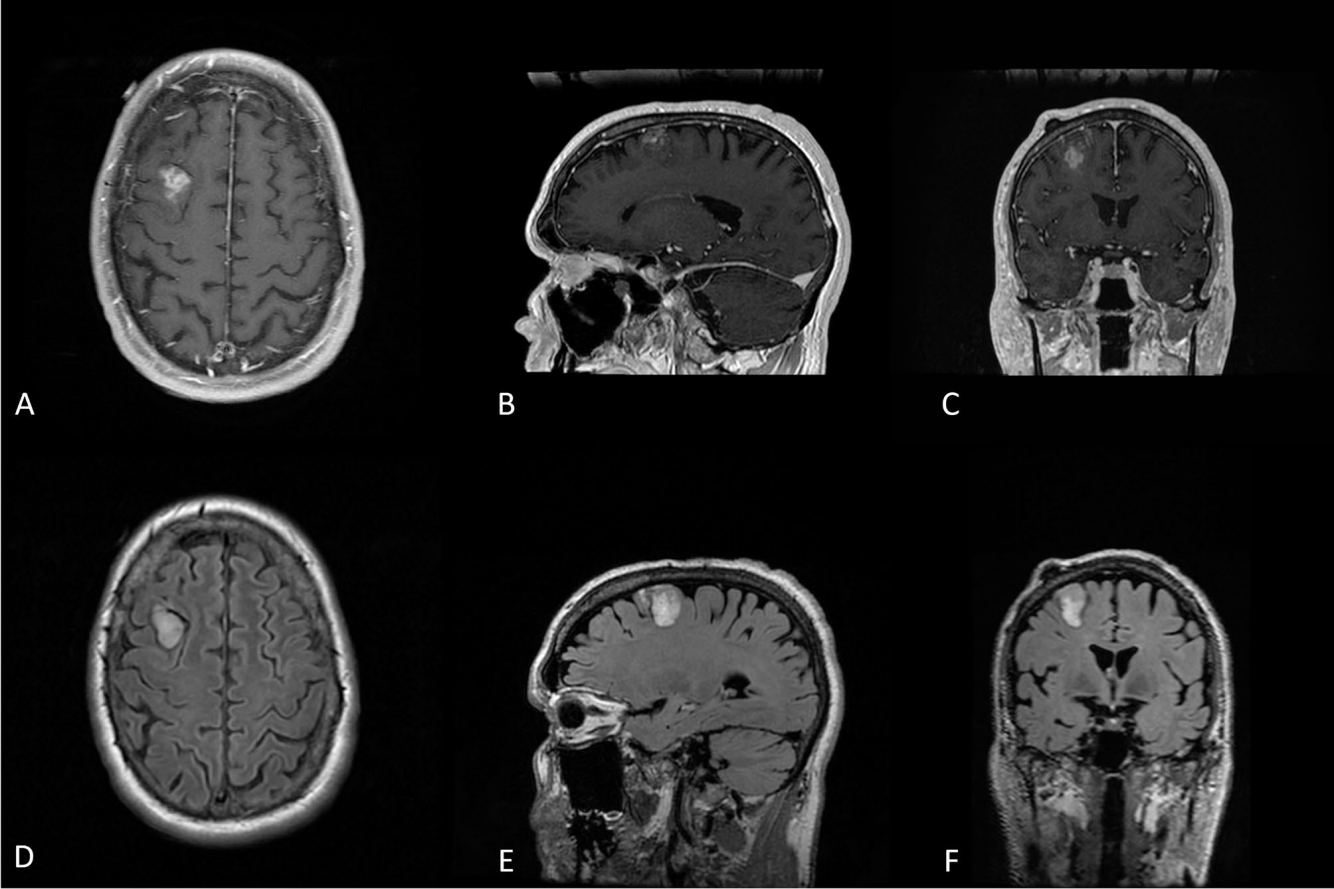
Axial (**A**), sagittal (**B**), and coronal (**C**) cuts of a T1-weighted post-contrast MRI and axial (**D**), sagittal (**E**), and coronal (**F**) cuts of a T2 FLAIR MRI brain in a 62 year old man with an incidentally discovered mass in the right middle frontal gyrus. The post-contrast images in **A-C** demonstrate serpiginous enhancement. **D-F** highlight FLAIR hyperintensity without surrounding vasogenic edema.An unrelated osteoma can also be seen overlying the mass

**Fig. 2:**
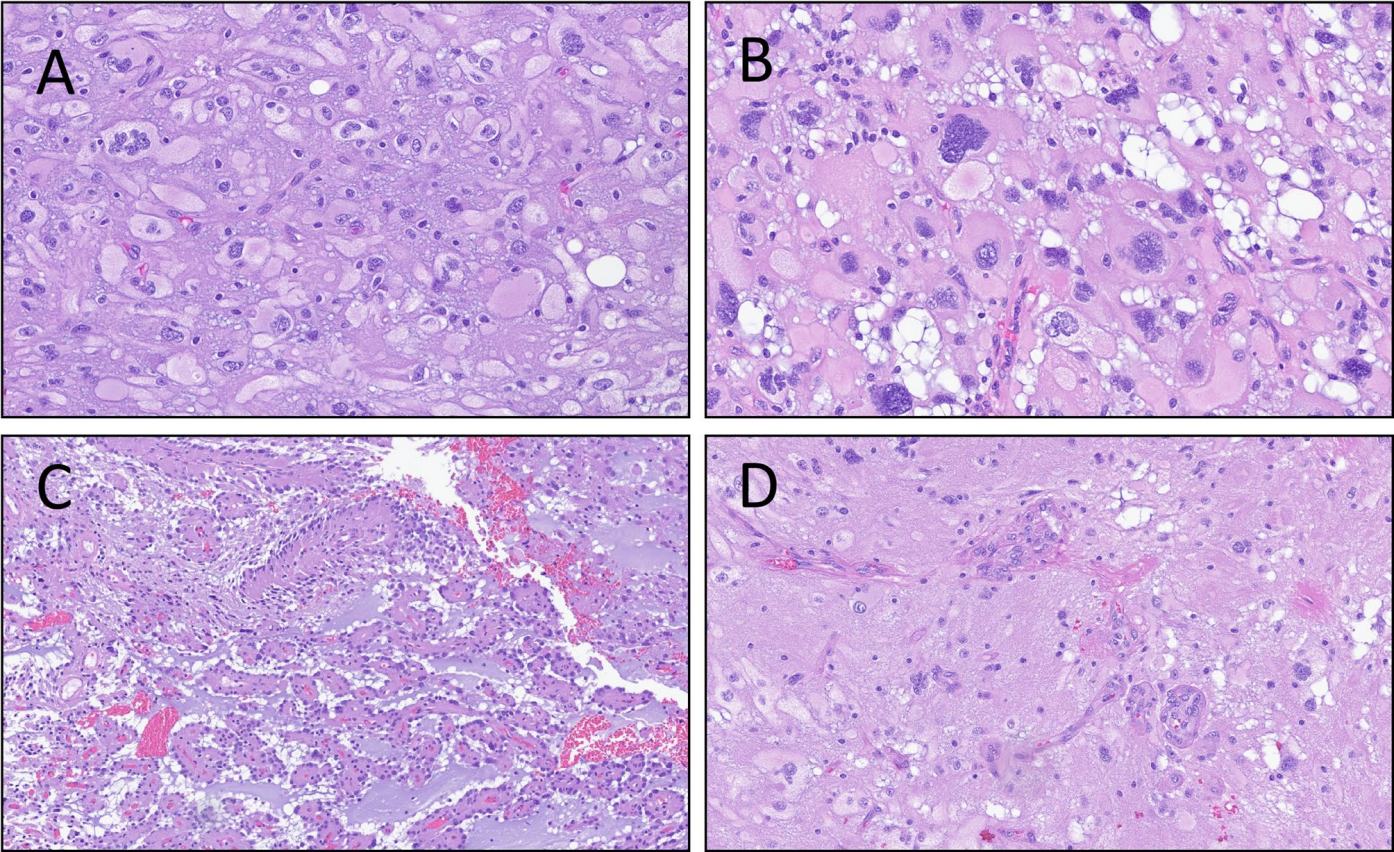
Representative histomorphologic images of tumor 1 showing (**A**), cells with abundant glassy eosinophilic cytoplasm, (**B**), bizarre atypia, (**C**), foci of papillary architecture, and (**D**), microvascular proliferation

The patient is a 62-year-old white male who was in his usual state of health prior to presentation (Table 1). He underwent cranial imaging after a motor vehicle collision where his car was struck by a large truck and was incidentally found to have a 2.8 cm, heterogeneously enhancing and well-circumscribed right frontal mass with a small cystic area without surrounding vasogenic edema (see Fig. 1A-F). Advanced brain tumor imaging studies were performed. The tumor showed intrinsic T1 hyperintensity, suggestive of calcifications or hemosiderin. An intermediate signal was detected via fluid attenuated inversion recovery (FLAIR) imaging. Diffusion-weighted MRI (DWI) showed restricted diffusion, suggestive of increased cellularity. Dynamic susceptibility contrast (DSC) and arterial spin labeled (ASL) studies showed relative increased blood flow, supporting a higher grade neoplasm. Magnetic resonance spectroscopy (MRS) revealed an elevated choline to creatine ratio with decreased N-acetylaspartate (NAA), also supporting a neoplastic process. Due to the circumscription of the neoplasm, a glioblastoma was thought to be unlikely and diagnostic considerations included a high grade ependymoma or oligodendroglioma. His exam failed to reveal any neurologic deficits. The patient was scheduled for surgery and underwent a gross total resection. He was discharged on postoperative day 2 after an uneventful hospital course. H&E stained sections showed a circumscribed glial neoplasm with foci demonstrating large, atypical cells with significant pleomorphism and glassy eosinophilic cytoplasm (Fig. 2A, B). Other areas showed a papillary architecture (Fig. 2C). Microvascular proliferation (Fig. 2D) and readily apparent mitotic activity were noted. The tumor cells were positive for GFAP and negative for IDH1-R132H with retained nuclear expression of ATRX (wildtype expression) and strong diffuse p53 expression, indicative of underlying *TP53* mutation. NGS showed *PBRM1* and *TP53* mutations. DNA methylation profiling revealed a strong consensus match to HPAP (Bethesda classifier version 3.0, 0.995) with loss of chromosome 13 noted on copy number plot. Versions 11b6 and 12b6 of the Heidelberg classifier revealed scores of 0.33 and 0.5 respectively to the class of pleomorphic xanthoastrocytoma. He was managed expectantly, but magnetic resonance imaging (MRI) three months after surgery revealed early recurrence. The patient underwent 57 Gy proton radiation in 30 fractions, completed approximately four months after surgery. He has not undergone systemic treatment. The patient was last seen 20 months after surgery; he was asymptomatic with no imaging evidence of recurrence.

**Table 1:** Case data

	Age/Sex^1^	Molecular Findings	Tumor Location	Surgery^2^	Postoperative KPS^3^	Radiation Dose (cGy)	Systemic Treatment	Progression-free Survival (months)	Treatment for Progression	Overall Survival (months)
1	62/M	*PBRM1* p.R519fs, *TP53* p.R273H, monosomy 13	Right frontal	GTR	90	57	None	2.73	Radiation	n/a
2	62/F	*FGFR1* p.I545Sfs*87, *TERT* promoter c.-146 C > T; monosomy 13; *MGMT* promoter unmethylated	Right parietal	Biopsy->GTR	90	59.4	None	86.53	Re-resection and re-irradiation	n/a
3	25/F	*RB1* p.R552* and *TP53* p.V143A; *EGFR* and *MET* amplification; chromosome 7 gain, chromosome 10, 13, and 17 loss	Left frontal	GTR	90	60	None	None	NA	n/a

^1^M= male, F = female; ^2^GTR= gross total resection; ^3^KPS= Karnofsky performance score

#### Case 2

**Fig. 3:**
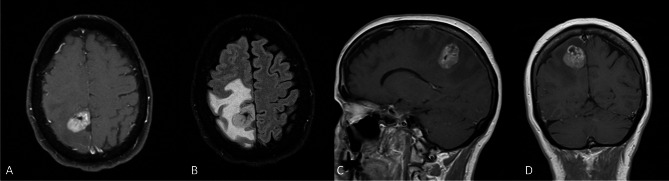
Axial T1 post-contrast image depicting a well-circumscribed, homogeneously enhancing right parietal mass in a 62 year-old woman with subjective left-sided weakness (**A**), axial T2 FLAIR image depicting significant perilesional edema resulting in global sulcal effacement in the ipsilateral cerebral hemisphere (**B**), and sagittal (**C**) and coronal (**D**) T1 post-contrast MRI also demonstrating a relatively circumscribed mass with enhancement and showing the location of the mass at the grey-white junction

**Fig. 4:**
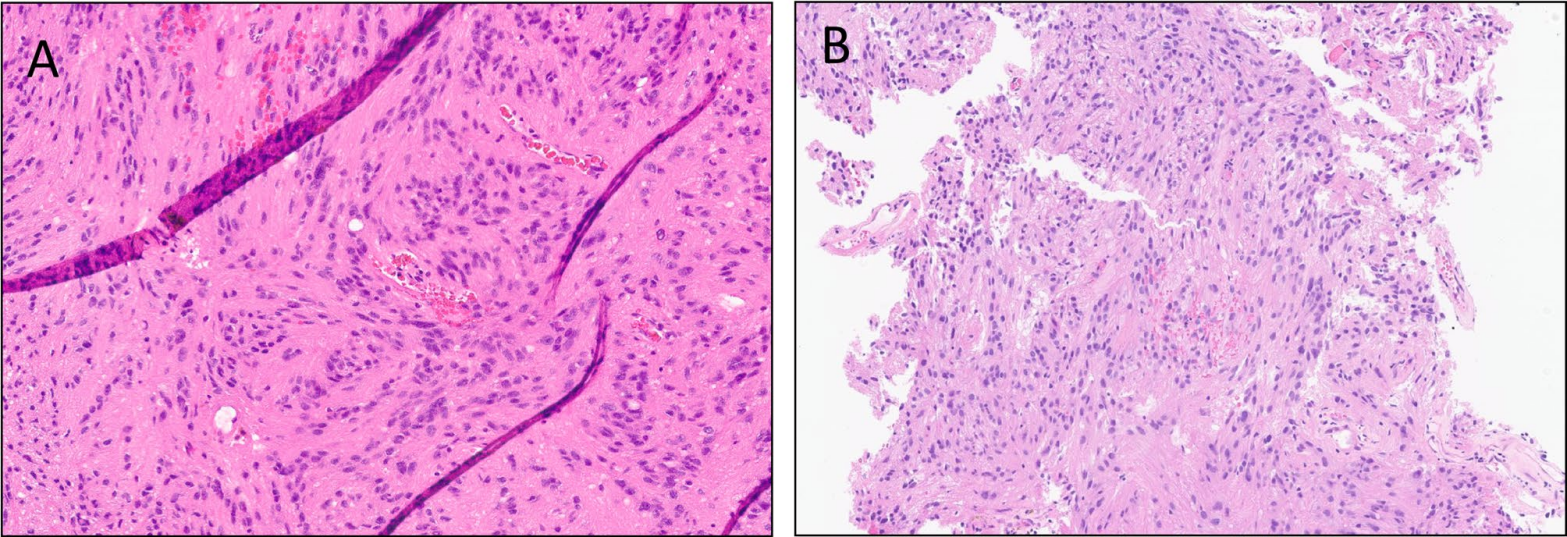
Representative histomorphologic images of tumor 2 showing (**A**), H&E stained section of the primary tumor showing ovoid tumor cells with fibrillary cytoplasm with some arranged around vessels (**B**), H&E stained section of the recurrent tumor showing similar morphology to the initial tumor with ovoid cells with stippled chromatin with fibrillary cytoplasm and vague rosette formation in the absence of overt high grade features

The patient is a 62-year-old white female with hyperlipidemia and remote 20 pack-year smoking history who presented to her primary care physician with several weeks of subjective left leg heaviness and three days of subjective left leg weakness (Table 1). Despite these symptoms, her neurologic exam revealed no deficits. An MRI revealed a 3.1 cm hemorrhagic enhancing right parietal mass with significant surrounding vasogenic edema out of proportion to the size of the mass (Fig. 3A-D). Differential diagnostic considerations included high grade glioma versus a metastatic lesion. CT chest/abdomen/pelvis did not reveal a primary malignancy and the former was favored. She underwent stereotactic biopsy of the lesion, which was suggestive of glioma. The patient therefore underwent gross total resection approximately two weeks later. She was discharged home on postoperative day 2. Pathology was consistent with grade II ependymoma based on 2007 WHO criteria (Fig. 4A) with immunohistochemistry showing positivity for GFAP, negativity for IDH1-R132H, and focal dot-like staining for EMA. The patient completed radiation therapy with 59.4 Gy in 33 fractions approximately 7 weeks after resection. Seven years after her initial surgery, the patient was noted to have recurrence on routine imaging. Three months later, the patient underwent repeat resection of her tumor followed by re-irradiation with 40 Gy in 20 fractions, completed approximately four months later. H&E stained sections of this recurrence once again showed features of an ependymal neoplasm without overt infiltration into adjacent parenchyma or high grade histologic findings (Fig. 4B). No discrete papillary architecture was noted and no bizarre cellular atypia was observed. NGS was performed on this recurrence and alterations were identified in *FGFR1*and the *TERT* promoter with monosomy of chromosome 13 (including copy number loss of *RB1*). Though no NGS was performed on the prior specimen to compare, the tumors looked morphologically identical. DNA methylation profiling was performed which revealed a high confidence score (0.94, Bethesda classifier version 2) to HPAP. Versions 11b6 and 12b6 of the Heidelberg classifier showed low scores for ependymoma (0.11 and 0.15 respectively). The patient was last seen approximately 9 years following her initial and 1.5 years following her subsequent resection, with only post-treatment changes noted on MRI. She remains on imaging surveillance and has not received any systemic treatment.

#### Case 3

**Fig. 5:**
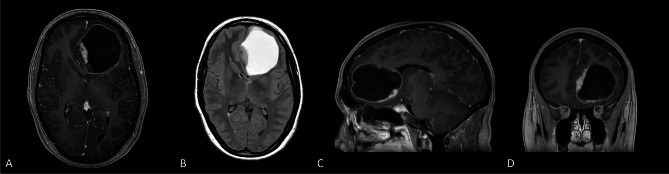
Axial T1 post-contrast (**A**), axial T2 FLAIR (**B**), and sagittal (**C**) and coronal (**D**) T1 post-contrast MRI brain demonstrating a large, well-circumscribed cystic left frontal mass with an enhancing nodule situated on the inferomedial aspect in a 25 year old woman with headaches and blurry vision. Despite local midline shift and considerable mass effect, there is little perilesional edema. On FLAIR (**B**), the cyst contents are hyperintense to CSF, likely reflecting higher protein content

**Fig. 6:**
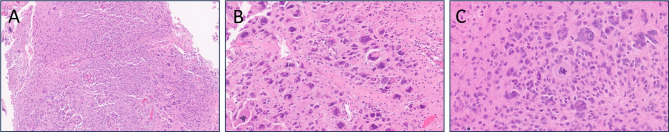
Representative histomorphologic images of tumor 3 showing (**A**), vague perivascular arrangement of tumor cells and foci of microvascular proliferation, (**B**), highly pleomorphic tumor cells, and (**C**), mitotic activity, including an atypical mitotic figure

The patient is a 25-year-old white female with no significant medical history who presented to an outside institution with headaches and blurry vision (Table 1). She was found to have papilledema in her left eye and underwent an MRI, which demonstrated a cystic, 6 cm left frontal mass with peripheral enhancing nodule showing FLAIR hyperintensity without surrounding vasogenic edema (Fig. 5A-D). Radiologic diagnostic considerations included pilocytic astrocytoma, ganglioglioma, and pleomorphic xanthoastrocytoma. She underwent gross total resection and had an uneventful postoperative course. H&E-stained sections showed a circumscribed glioma with large, highly atypical cells, mitotic activity, and vascular proliferation (Fig. 6A-C). NGS revealed mutations in *RB1* and *TP53*, loss of chromosomes 10, 13, and 17, gain of chromosome 7, and *EGFR* and *MET* amplification. DNA methylation profiling showed a suggestive match to the class of HPAP (Bethesda classifier version 3.0, 0.746). Though this score was slightly below the clinical threshold of 0.8, it is thought that the relatively low score is due to low tumor purity. Importantly, this tumor did not show a match to the class of glioblastoma (versions 11b6 and 12b6 of the Heidelberg classifier revealed scores of 0.44 and 0.4 respectively to low grade glioma/ganglioglioma) and shows other molecular features consistent with HPAP. The patient completed radiation therapy with 60 Gy in 30 fractions approximately three months after surgery. Approximately five months from surgery, the patient remains alive and progression-free.

**Fig. 7:**
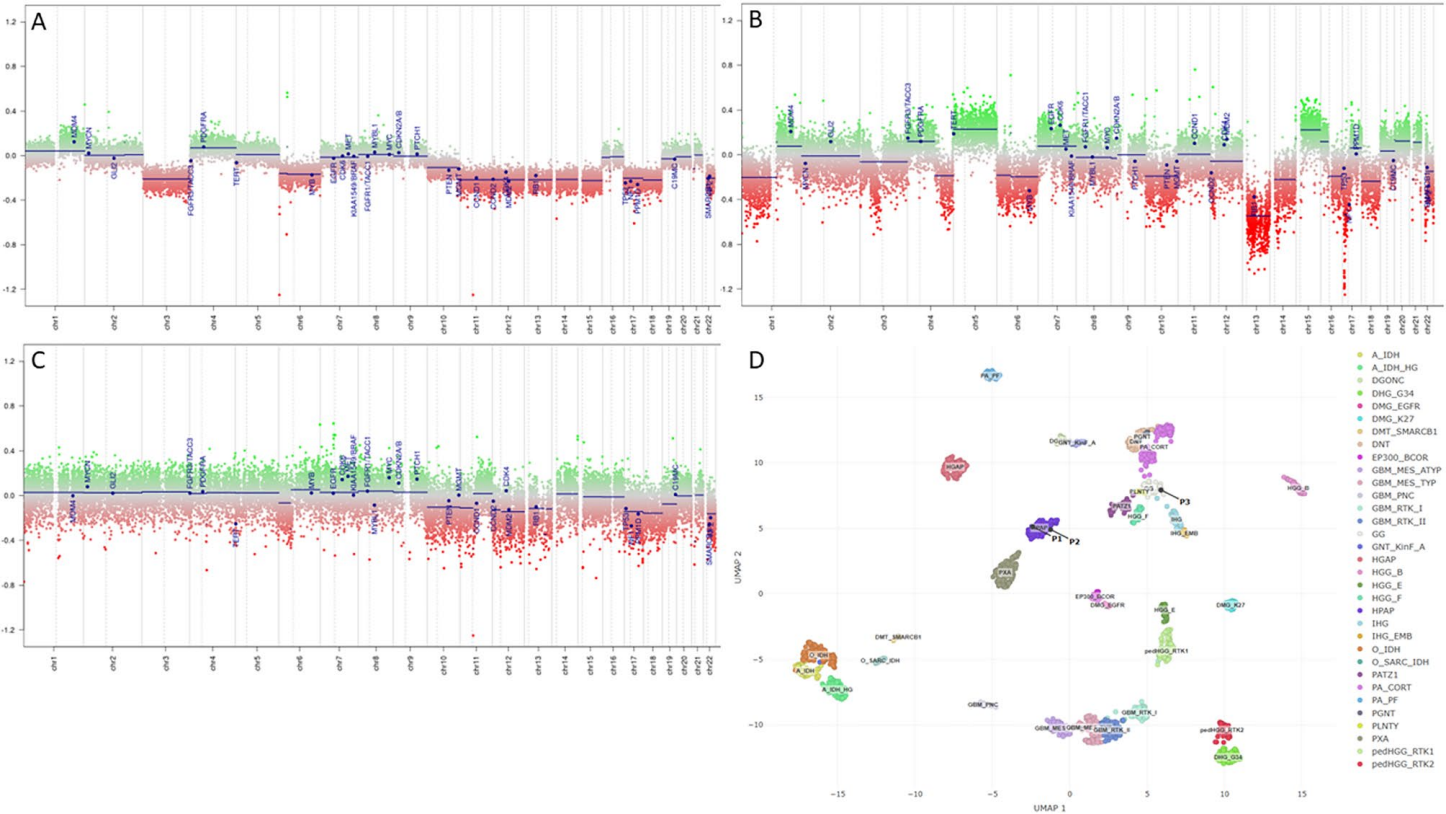
Copy number plots of case 1 (**A**), case 2 (**B**), and case 3 (**C**) showing the common loss of chromosome 13. (**D**), Uniform Manifold Approximation and Projection (UMAP) showing the three cases (P1: patient 1, P2: patient 2, P3: patient 3). Note that P3 clusters with ganglioglioma; this case had the lowest score (0.746) and the relatively low score and clustering on UMAP is thought to be due to low tumor content. Of note, this tumor does not cluster near glioblastoma

A summary of the copy number plots for each case and a composite Uniform Manifold Approximation and Projection (UMAP) is provided in Fig. 7.

## Discussion

The increasing use of modern molecular diagnostic techniques, particularly the utilization of DNA methylation profiling to categorize tumors based on epigenetic profile, has shifted the paradigm in terms of CNS tumor classification. The methylation profile of HPAP most closely resembles pleomorphic xanthoastrocytoma (PXA) and polymorphous low grade neuroepithelial tumor of the young (PLNTY) yet remains distinct from both and, importantly, is distinct from GBM [4–6]. Though HPAP shares features with other tumor types, the relatively circumscribed nature of the tumor along with specific molecular clues, particularly loss of chromosome 13 and mutations in *RB1* and *TP53*, should prompt one to consider this entity, especially given its propensity to harbor a better clinical prognosis when compared with other high grade glial neoplasms. The results of our study are largely in keeping with the existing literature. Our patient cohort was older than that described by Pratt [4]though this likely reflects our small sample size. Similar to initial reports, our patients had a wide variety of radiographic and histologic presentations, although all tumors in our series were well-delineated from adjacent brain parenchyma without overt evidence of infiltration. All cases demonstrated monosomy of chromosome 13, two showed *TP53* mutations and 2 showed *RB1* mutations. Interestingly, one patient (patient 3) showed gain of chromosome 7 and loss of chromosome 10 with amplification of *EGFR*, features considered diagnostic of GBM. However, the current WHO criteria states that GBM must be a diffuse glioma and this patient’s tumor was very well-circumscribed both on imaging and following histopathology examination. Close follow-up will be necessary to see the evolution of this tumor, but thus far the clinical course is more in line with HPAP, despite this molecular signature. Of interest, a GBM-like molecular signature has also been described in a subset of HPAP by Pratt et al. [4]

Given the rarity of this tumor type, no standard treatment has been established. While surgical resection remains of utmost importance, management after surgery is still poorly understood. Though radiation was used for all patients in our study, it is not clear that it is required; if it is required, the timeline by which it should be used is similarly murky as there is currently no longterm data for whether early or late radiation affects the natural progression of this tumor.

The low rate of *MGMT* promotor methylation seen in HPAP likely confers resistance to traditional alkylating agents like temozolomide [4]. Mutations in the tumor suppressor *RB1*, found in approximately one fourth of HPAP [4], are common in other malignancies like retinoblastoma, melanoma, and osteosarcoma [7]. RB1 deficiency confers several specific vulnerabilities to targeted therapies that have shown promise in clinical and preclinical study. For example, in vitro evaluations of LY3295668 and ENMD-2076, both Aurora kinase inhibitors, have been effective against *RB1*-mutant small cell lung cancer cells [8, 9], and LY3295668 was effective at treating RB1-deficient retinoblastoma in vivo [10]. Preclinical evaluation of PARP inhibitors have also shown promise in multiple RB1-deficient cancers such as lung adenocarcinoma and osteosarcoma [11, 12]. 

Several other potential targets exist in HPAP. *BRAFV600E* mutations, seen in 12% of HPAP [4]are also potential therapeutic targets when present. BRAF inhibitors are currently approved for the treatment of several other malignancies including melanoma and non-small cell lung carcinoma [13]. One patient in our study had an *FGFR1* mutation, more commonly seen in HGAP [1, 4]. FGFR inhibitors have shown great promise in treating various malignancies including breast, gallbladder, and lung cancers [14–16]. Future study is warranted to identify the best course of treatment. All patients at our institution have been treated solely with radiation. This fact is an important consideration, particularly for younger patients diagnosed with HPAP as lack of chemotherapy can spare them fertility preservation. Given the spectrum of molecular alterations seen in this entity, personalized targeted therapy could be considered on an individual tumor basis.

HPAP has been shown to have survival outcomes roughly equal to those observed in PXA and HGAP and superior to those seen in GBM—even in tumors with GBM-associated features like microvascular proliferation or necrosis [4, 5]. While extrapolating from our series is difficult given our small sample size, the patients reported do seem to fare better than patients with GBM, although the early recurrence in patient 1 suggests that these tumors can display aggressive behavior and further follow-up is warranted before definitive conclusions can be made.

Overall, we share evidence that supports HPAP as a distinct entity from other high grade gliomas like GBM. We acknowledge that there is some variability from patient to patient in terms of clinical and histopathologic presentation; however, there is inherent variability even in currently acknowledged diagnostic entities. Surely as more HPAP are reported in the literature a more detailed clinical, radiologic, and pathologic description will emerge. All that said, these tumors do share common features including radiologic and histopathologic circumscription, typically either an ependymal or papillary architectural pattern with most showing overt pleomorphism, and common molecular findings to include recurrent loss of chromosome 13, *TP53* mutations, and a unique DNA methylation profile. Given the better overall survival, even in the absence of chemotherapy in our series, we are advocates for splitting this group into a separate diagnostic class and emphasize our findings as a starting point to recognize this entity in a diagnostic work-up.

### Limitations

The primary limitations of our study are the small sample size, limited follow-up, and the retrospective nature of our report. This obfuscates our inferential power and any statistical analysis or determination of appropriate treatment strategies. Similarly, the rare nature of HPAP leads patients to seek multiple opinions, so some data in our report are incomplete. Further study with larger series is needed to better characterize HPAP and the optimal treatment for this very rare tumor.

## Conclusions

HPAP is a diagnostic product of modern molecular characterization techniques. It has a widely variable histologic and radiographic appearance, sharing features with both high- and low-grade tumors, and its prognosis is accordingly intermediate. Further study is needed to fully characterize this entity and its optimal treatment, though the limited data available in the literature support this tumor type as a distinct entity with a favorable clinical course.

## Data Availability

No datasets were generated or analysed during the current study.
